# Object recognition and localization enhancement in visual prostheses: a real-time mixed reality simulation

**DOI:** 10.1186/s12938-022-01059-7

**Published:** 2022-12-24

**Authors:** Reham H. Elnabawy, Slim Abdennadher, Olaf Hellwich, Seif Eldawlatly

**Affiliations:** 1 Digital Media Engineering and Technology Department, Faculty of Media Engineering and Technology, German University in Cairo, Cairo, Egypt; 2 Computer Science and Engineering Department, Faculty of Media Engineering and Technology, German University in Cairo, Cairo, Egypt; 3 Computer Science Department, Faculty of Informatics and Computer Science, German International University, New Administrative Capital, Egypt; 4 Chair of Computer Vision and Remote Sensing, Technische Universität Berlin, Berlin, Germany; 5 Computer and Systems Engineering Department, Faculty of Engineering, Ain Shams University, 1 El-Sarayat St., Abbassia, Cairo, Egypt; 6 Computer Science and Engineering Department, The American University in Cairo, Cairo, Egypt

**Keywords:** Simulated prosthetic vision, Object recognition, Object localization, Real-time mixed reality simulation

## Abstract

Blindness is a main threat that affects the daily life activities of any human. Visual prostheses have been introduced to provide artificial vision to the blind with the aim of allowing them to restore confidence and independence. In this article, we propose an approach that involves four image enhancement techniques to facilitate object recognition and localization for visual prostheses users. These techniques are clip art representation of the objects, edge sharpening, corner enhancement and electrode dropout handling. The proposed techniques are tested in a real-time mixed reality simulation environment that mimics vision perceived by visual prostheses users. Twelve experiments were conducted to measure the performance of the participants in object recognition and localization. The experiments involved single objects, multiple objects and navigation. To evaluate the performance of the participants in objects recognition, we measure their recognition time, recognition accuracy and confidence level. For object localization, two metrics were used to measure the performance of the participants which are the grasping attempt time and the grasping accuracy. The results demonstrate that using all enhancement techniques simultaneously gives higher accuracy, higher confidence level and less time for recognizing and grasping objects in comparison to not applying the enhancement techniques or applying pair-wise combinations of them. Visual prostheses could benefit from the proposed approach to provide users with an enhanced perception.

## Introduction

Vision is considered the most important sense that any human cannot live without independently. Loss of vision has been demonstrated to hinder the independence and the confidence for any human being. This loss of vision may occur due to some diseases such as retinitis pigmentosa that affects the peripheral vision or aged-macular degeneration that affects the central vision [1]. These diseases damage the photoreceptors in the eye retina, where the rods and the cones are no more able to convert light energy into electrical signals to the brain which causes the loss of vision [2]. As a result, visual prostheses were introduced to provide a solution, through artificial vision, for partial restoration of the lost vision by means of electrically stimulating the visual pathway [3]. Different types of visual prostheses have been introduced including retinal implants, thalamic visual prostheses and cortical visual prostheses [4]. Retinal prostheses, considered the most successful so far, could be epiretinal such as Argus II, subretinal such as Alpha IMS or suprachoroidal such as Bionic Vison Australia [5]. In epiretinal prosthesis, the electrodes are implanted near the ganglion cells and nerve fibers [6]. Subretinal prostheses have closer proximity to the natural circuits of the retina. In suprachoroidal prosthesis, the electrodes are implanted between the sclera and the choroid [7]. The mechanism of the system works as follows: a tiny camera is mounted at the middle of an eye glass that captures the image, sends the image through wired connection to a video processing unit, where the conversion to electrical stimulation signals is performed. Then, the stimulus is transferred wirelessly to the implant enabling users to perceive patterns of light [8].

Prosthetic vision comprises spots of light called phosphenes that are perceived by the implanted patient [9]. Each phosphene maps to one electrode available in the implant. A limitation in visual prosthetic devices is the limited number of electrodes in the implant, which results in a low spatial resolution, that hinders the full perception of the visual scene [10]. Such limitation could be alleviated by increasing the number of electrodes, which is expected to enhance the spatial resolution of the images perceived via visual prostheses [11, 12]. However, the perceived image will remain far from the image perceived by normally sighted individuals. Another limitation is the limited number of gray levels available through these devices to represent the perceived image, which affects preserving the details in the image [12]. This could be solved via increasing some system-level constraints and restrictions such as wireless transmission bandwidth and processing capabilities of the implanted module so that the number of gray levels can be increased [13, 14]. In addition, electrodes malfunctioning might happen over time causing dropouts at the corresponding location in the visual field [15]. This dropout can be handled by translating the object of interest to a location in the visual field that contains the minimum number of dropouts for better visualization and recognition [16].

Previous studies that used image enhancement techniques focused on objects segmentation to retrieve each object, either in an image with multiple objects or a single object, at a time and display it in phosphene simulation. However, these segmented objects remain with their details which complicates the object representation when displayed through visual prostheses [17]. Moreover, contrast enhancement was suggested in previous studies to enhance object recognition [18]. Despite its potential enhancement, it might not help in providing a clear representation of the objects especially when the scene is a complex one. Furthermore, wavelet-based image processing techniques were addressed to enhance the recognition in the low-resolution environment [19]. However, this might also not provide clear representation of the images since the objects remain with their details.

Given the poor spatial and radiometric resolutions of current visual prostheses system, that gets more problematic with the existence of electrode dropouts, we propose a system for enhancing object localization and object recognition. Object recognition refers to the ability of the users to correctly identify an object’s identity, whereas object localization refers to the ability of the users to correctly identify the location of an object and grasp it. The first enhancement is the usage of clip art representation in place of the actual object as a scene simplification method to allow better recognition. Second, we utilize an edge enhancement technique along with corners enhancement to sharpen the edges of the object for better detail preservation. While edge enhancement techniques were examined before in the context of enhancing prosthetic vision [20], they were not combined with using clip art representation. Finally, we apply a dropout handling technique to support preserving the maximum possible numbers of phosphenes despite the existence of the malfunctioned electrodes that caused dropout [16]. The proposed approach is examined in a mixed reality environment that simulates perceived vision by visual prostheses users.

## Results

### Enhancement techniques and phosphenes simulation outcome

**Fig. 1:**
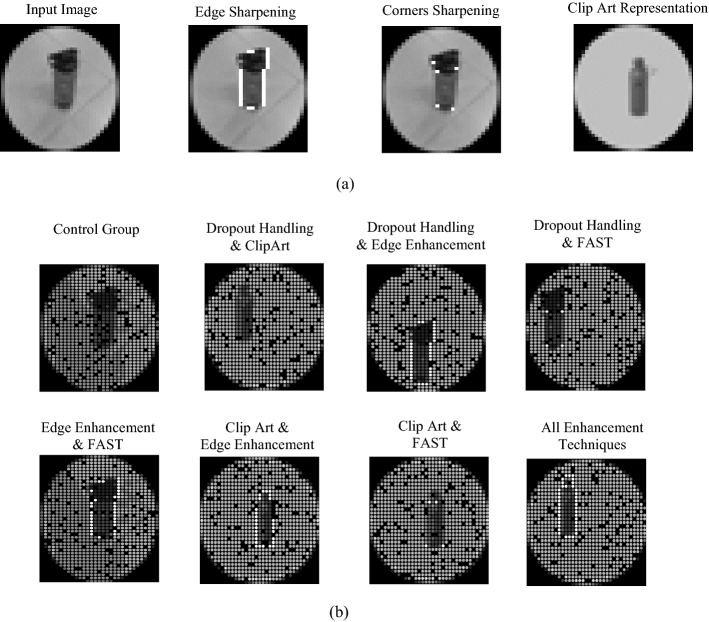
Single-object experiments. **a** Enhancement techniques. **b** Phosphene simulation of the 8 experiments

To illustrate the shape of the image after applying edge sharpening, FAST and clip art representation, Fig. 1a shows the input image after applying each of the three methods. It can be observed that the edges are sharpened surrounding the bottle making it easier in recognition due to the outline given to the bottle that resulted from the edge enhancement. Applying FAST emphasized the corners at the bottle’s neck which could enable easier identification of the bottle. Finally, using the clip art representation simplifies the bottle further, which in combination with other enhancement techniques is expected to enhance the ability of visual prostheses users to recognize the presented objects.

We next demonstrate the difference between the outcomes obtained from the single objects experimental setups as shown in Fig. 1b, where the outcomes are shown for different participants. As it is shown, the result from the “Control Group” experiment illustrates what the control group participants see where the real object (i.e., bottle) is barely recognized due to the dropout existence that overlaps with the bottle’s body. The utilization of dropout handling and clip art can be shown in the “Dropout Handling and Clip Art” experiment, where the real bottle is replaced by its clip art and translated to the best location within the visual field that has the minimum dropped out locations. Moreover, it can be observed that the addition of the dropout handling and edge enhancement to the real object, as shown in “Dropout Handling and Edge Enhancement” experiment, demonstrates that the edges of the bottle are sharpened and the real bottle is translated to the best location within the visual field with minimum dropouts. Furthermore, applying dropout handling and FAST, as demonstrated in the “Dropout Handling and FAST” experiment, highlights corners at the neck of the bottle in addition to the translation of the real bottle to the location with minimum dropout. In addition, further enhancement to the real bottle can be perceived when utilizing edge enhancement and FAST to the bottle, as indicated in the “Edge Enhancement and FAST” experiment, where the edges of the bottle along with its corners are sharpened. This enhancement was further improved when using the clip art of the bottle along with edge enhancement, as revealed in the “Clip Art and Edge Enhancement” experiment, where the clip art of the bottle along with the edges of the clip art are sharpened as shown. Similarly, the usage of clip art along with FAST, as shown in the “Clip Art and FAST” experiment, sharpens essential corners pixels in the bottle. Finally, the utilization of all the aforementioned enhancement techniques, as illustrated in the “All Enhancement Techniques” experiment, shows the best possible look for any arbitrary object enhancing objects recognition. This was shown when the real bottle was replaced by its clip art representation in addition to sharpening both the edges and the corners of the clip art bottle along with translating the bottle to the place with minimum dropout. The figure demonstrates that the usage of the clip art of the bottle gave better visualization of the bottle where the real bottle was confusion in recognition.

**Fig. 2:**
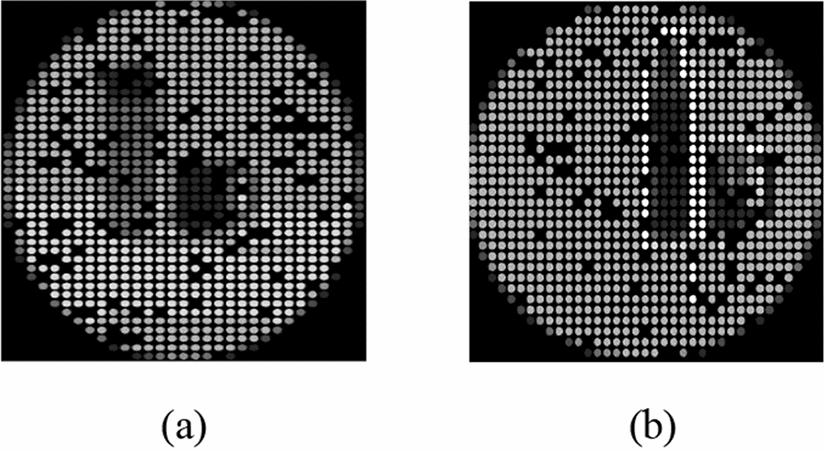
Multiple-object experiments. **a** Control group. **b** All enhancement techniques

**Fig. 3:**
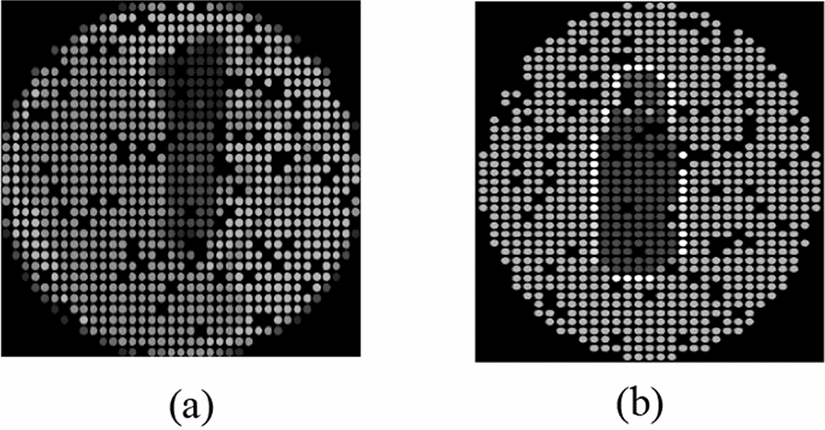
Navigation experiments. **a** Control group. **b** All enhancement techniques

To demonstrate the outcomes from the multiple objects grouping experiments, Fig. 2 shows the outcomes from the two experiments conducted in the multiple objects setup, obtained from two different participants. The “Control group” experiment contains the real bottle and cup without any enhancement technique applied. However, the “All Enhancement Techniques” experiment shows the phosphene simulation of the bottle and the cup after replacing each of the two objects by their corresponding clip art along with sharpening both the edges and the corners in addition to translating the objects to the location with minimum dropout. Similarly, Fig. 3 shows the outcomes from the two experiments utilized in the navigation system setup for two different participants. The “Control Group” experiment shows the real bottle in the phosphene simulation without the application of any enhancement technique, whereas the “All Enhancement Techniques” experiment shows the phosphene simulation after sharpening the edges and the corners of the clip art object translated to the location with minimum dropouts.

### Experimental results

#### Single-object experiments

**Fig. 4:**
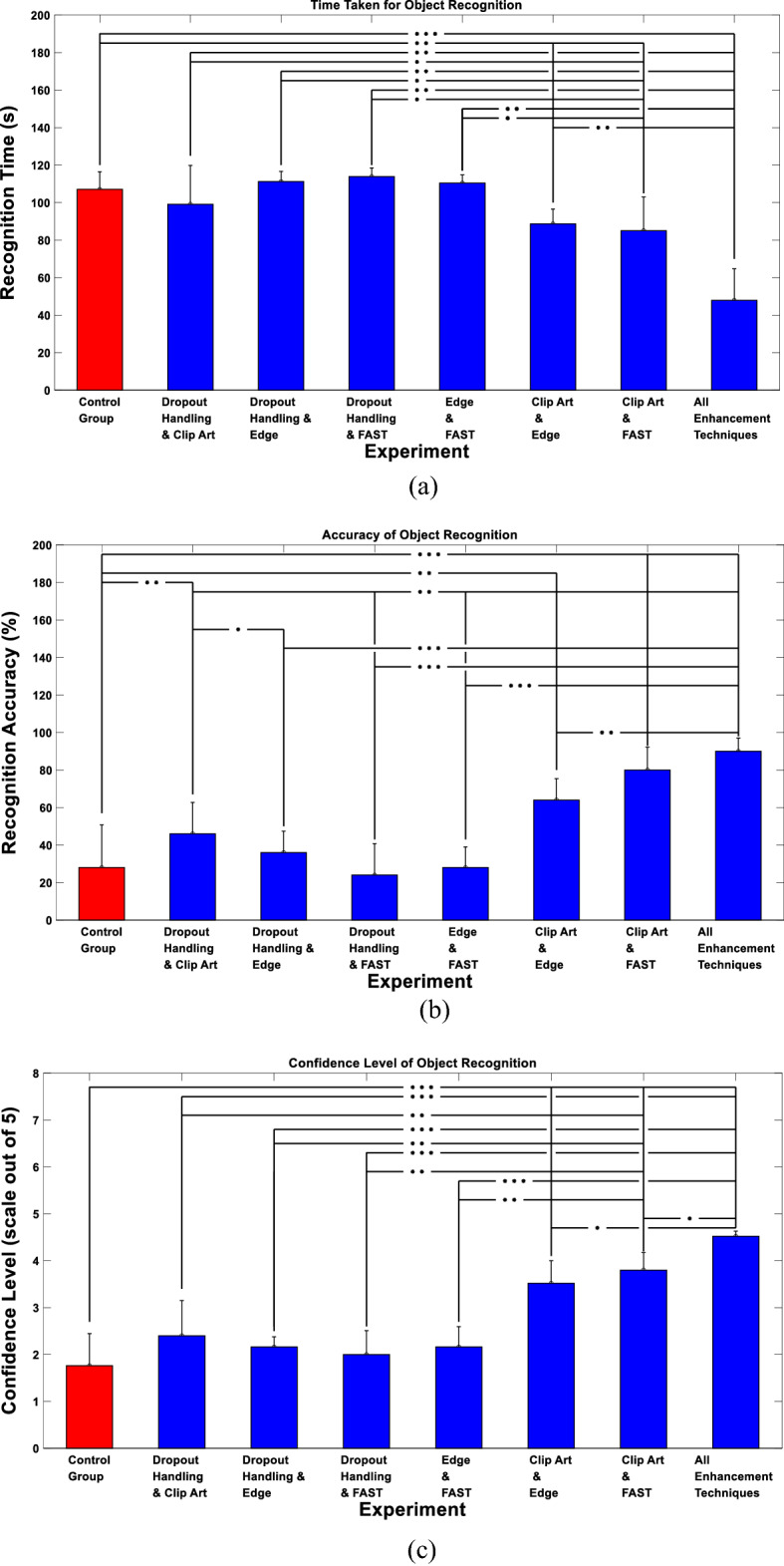
Single objects recognition results. **a** Recognition time. **b** Recognition accuracy. **c** Confidence level. *P < 0.05, **P < 1e−04, ***P < 1e−07, two-sample Wilcoxon test

We first examined the performance of the proposed enhancements when presenting the subjects with single objects. The 8 performed experiments were given the following names: Control Group, Dropout Handling and Clip Art, Dropout Handling and Edge Enhancement, Dropout Handling and FAST, Edge Enhancement and FAST, Clip Art and Edge Enhancement, Clip Art and FAST and, finally, All Enhancements Techniques. Figure 4a demonstrates the average time taken to correctly recognize the objects computed across all subjects participating in each experiment. The figure shows that the experiment with all enhancement techniques gives the least recognition time compared to the other experiments (Control Group: 107.08 ± 9.38 s, Dropout Handling and Clip Art: 99.12 ± 20.73 s, Dropout Handling and Edge Enhancement: 111.24 ± 5.43 s, Dropout Handling and FAST: 113.88 ± 4.58 s, Edge Enhancement and FAST: 110.4 ± 4.38 s, Clip Art and Edge Enhancement: 88.68 ± 7.88 s, Clip Art and FAST: 85.04 ± 17.99 s, and All Enhancement Techniques: 47.88 ± 16.84 s). Figure 4b demonstrates the accuracy of correctly recognized objects. The figure illustrates that the highest accuracy was also achieved in the experiment with all enhancement techniques compared to the other experiments (Control Group: 28 ± 22.8%, Dropout Handling and Clip Art: 46 ± 16.73%, Dropout Handling and Edge Enhancement: 36 ± 11.4%, Dropout Handling and FAST: 24 ± 16.73%, Edge Enhancement and FAST: 28 ± 10.95%, Clip Art and Edge Enhancement: 64 ± 11.4%, Clip Art and FAST: 80 ± 12.25%, and All Enhancement Techniques: 90 ± 7.07%). Finally, Fig. 4c demonstrates the confidence level of the participants denoting how confident they were when they recognized the object. Consistent with the recognition time and accuracy results, the figure shows that the experiment with all enhancement techniques gave the highest confidence level compared to the other experiments (Control Group: 1.76 ± 0.68, Dropout Handling and Clip Art: 2.4 ± 0.75, Dropout Handling and Edge Enhancement: 2.16 ± 0.22, Dropout Handling and FAST: 2 ± 0.51, Edge Enhancement and FAST: 2.16 ± 0.43, Clip Art and Edge Enhancement: 3.52 ± 0.48, Clip Art and FAST: 3.8 ± 0.37, and All Enhancement Techniques: 4.52 ± 0.11). To assess the significance of the results, two-sample Wilcoxon test was performed across all trials.

**Fig. 5:**
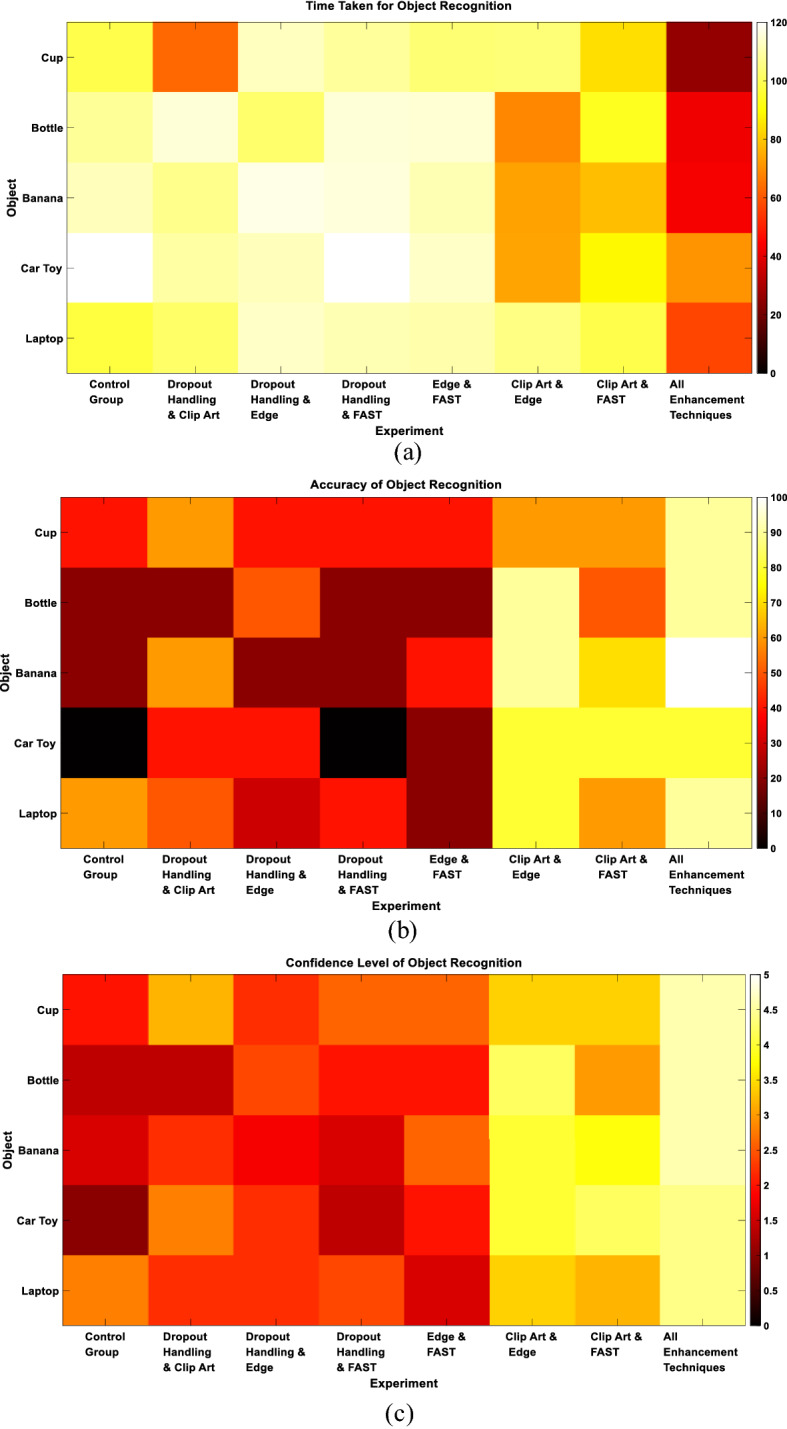
Single objects recognition performance for each displayed object. **a** Recognition time. **b** Recognition accuracy. **c** Confidence level

To further assess the ability of different approaches to enhance object recognition, we examined each of the three evaluation metrics (the time taken to recognize the object, the recognition accuracy and the confidence level) for each of the displayed objects. Figure 5 indicates that the banana was the easiest object to recognize, especially when clip art representation was used, achieving the best performance when all enhancement techniques were applied (recognition accuracy: 100%, average confidence: 4.6). This could be attributed to the distinct curved shape the banana has compared to the other objects. On the other hand, the car toy was relatively the hardest object to recognize across most of the examined techniques. However, its recognition was significantly enhanced when using all enhancement techniques compared to, for example, the control group (Control—Average Time Taken: 120 s, Recognition Accuracy: 0%, Average Confidence: 1; All enhancement—Average Time Taken: 70.8 s, Recognition Accuracy: 80%, Average Confidence: 4.4). In general, there was no object that was hard to recognize when using all enhancement techniques combined, which indicates that the proposed approach enhances the perception of these objects.

**Fig. 6:**
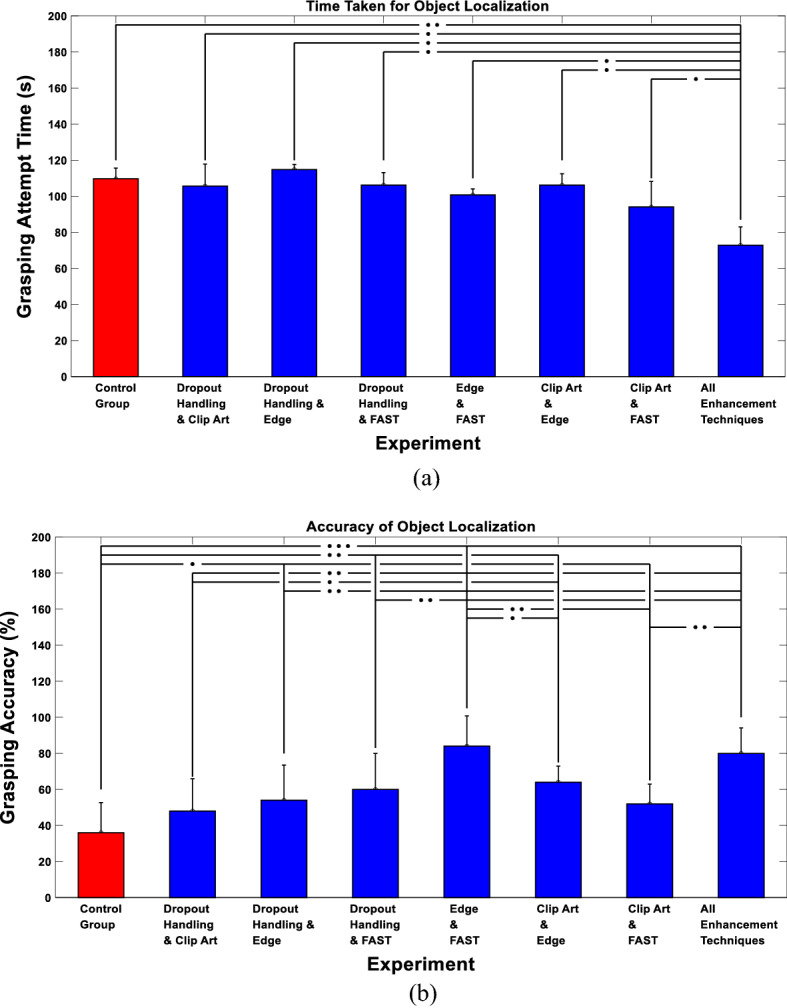
Single objects grasping results. **a** Grasping attempt time. **b** Grasping accuracy. *P < 0.05, **P < 1e−04, ***P < 1e–07, two-sample Wilcoxon test

We also examined the ability of the subjects to localize the objects using the proposed enhancements. Figure 6a shows the grasping attempt time of the participants demonstrating that the least time taken for the attempt to grasp an object is the experiment with all the enhancement techniques applied compared to the other experiments (Control Group: 109.68 ± 5.98 s, Dropout Handling and Clip Art: 105.68 ± 12.15 s, Dropout Handling and Edge Enhancement: 114.8 ± 2.8 s, Dropout Handling and FAST: 106.2 ± 6.94 s, Edge Enhancement and FAST: 100.72 ± 3.38 s, Clip Art and Edge Enhancement: 106.16 ± 6.29 s, Clip Art and FAST: 94.04 ± 14.17 s, and All Enhancement Techniques: 72.84 ± 10.13 s). In addition, Fig. 6b shows the accuracy of the participants in correctly grasping the object. The figure demonstrates that the experiments that contain Clip Art and Edge Enhancement (experiments 6 and 8), resulted in the highest grasping accuracy (Control Group: 36 ± 16.73%, Dropout Handling and Clip Art: 48 ± 17.89%, Dropout Handling and Edge Enhancement: 52 ± 17.89%, Dropout Handling and FAST: 60 ± 20%, Edge Enhancement and FAST: 84 ± 16.73%, Clip Art and Edge Enhancement: 64 ± 8.94%, Clip Art and FAST: 52 ± 10.95% and All Enhancement Techniques: 80 ± 14.14%). The results indicate that the proposed enhancement techniques preserved and simplified the details that describe the identity of the object.

**Fig. 7:**
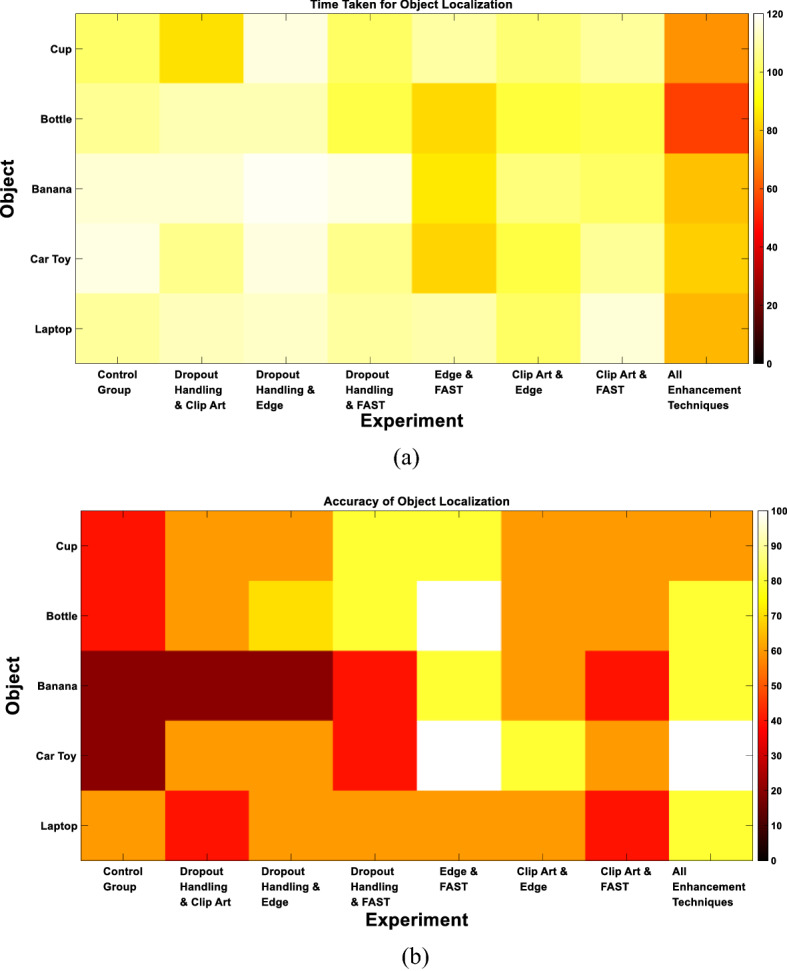
Single objects grasping performance for each displayed object. **a** Grasping attempt time. **b** Grasping accuracy

We finally assessed the performance achieved using different approaches with respect to the ability of the subjects to grasp each presented object. Figure 7 demonstrates that the car toy was the easiest to grasp compared to other objects, achieving an average grasping accuracy of 100% when all enhancement techniques were applied. Based on feedback from the subjects, the car toy was the easiest to grasp because of the wheels as they have a distinct circular shape that was easily located. On the other hand, despite the banana being the easiest to recognize, it was shown to be relatively the hardest to grasp. However, when all enhancement techniques were applied, and elevated grasping accuracy of 80% was achieved. This could be attributed to the fact that the banana was placed flat on the table, making it harder for the subjects to grasp. The figure also indicates that there is no direct correlation between the grasping attempt time and the grasping accuracy, which could be explained given the definition of the grasping attempt time that does not necessarily map to a correct grasp.

#### Multiple-object experiments

**Fig. 8:**
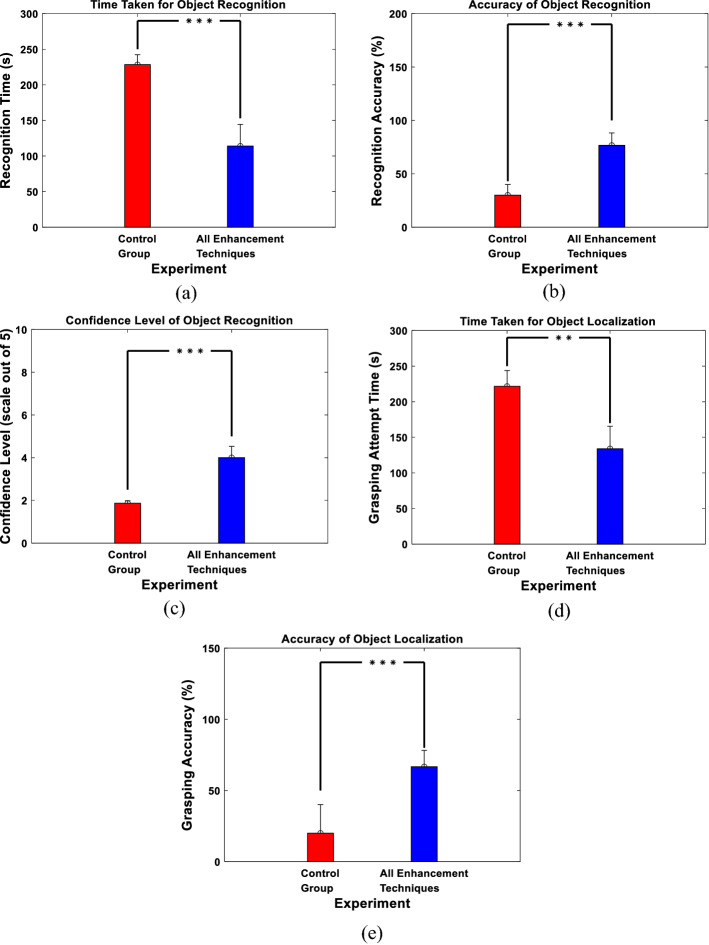
Multiple-object experiments results. **a** Recognition time. **b** Recognition accuracy. **c** Confidence level. **d** Grasping attempt time. **e** Grasping accuracy. **P < 1e−04, ***P < 1e−07, two-sample Wilcoxon test

In the second set of experiments, we examined the proposed approach in a more complex scene that contains multiple objects (a pair of presented objects) as opposed to single objects. Only two setups were examined: “Control Group” and “All Enhancement Techniques”, to compare the results obtained if no enhancement technique was used (i.e., the original real object utilized) to using all enhancement techniques in the phosphene simulation. The setup with “All Enhancement Techniques” was used since it resulted in the best performance in terms of object recognition and localization as observed in the single objects experiments. In terms of object recognition, Fig. 8a shows the time taken to correctly recognize the objects in the scene illustrating that the experiment with all enhancement techniques gave shorter period of time in objects recognition unlike that of the control group experiment (Control Group: 228.33 ± 14.01 s and All Enhancement Techniques: 113.93 ± 30.38 s). Figure 8b shows the accuracy of the correctly recognized objects out of the total objects. The figure shows that the experiment with all enhancement techniques also gave higher accuracy compared to that of the control group experiment (Control Group: 30 ± 10% and All Enhancement Techniques: 76.67 ± 11.55%). Finally, Fig. 8c shows the confidence level of the participants, where the experiment with all the enhancement techniques resulted in higher confidence level compared to that of the control group experiment (Control Group: 1.87 ± 0.12 and All Enhancement Techniques: 4 ± 0.53).

For object localization, Fig. 8d shows the time taken for grasping the objects correctly. The figure demonstrates, consistent with the object recognition experiments, that the participants of the experiment in which all the enhancement techniques were applied needed less amount of time for grasping the objects compared to the control group experiment (Control Group: 221.6 ± 22.2 s and All Enhancement Techniques: 133.93 ± 31.49 s). Figure 8e shows the participants accuracy in correctly grasping the objects, indicating higher accuracy when all the enhancement techniques are applied compared to that of the control group experiment (Control Group: 20 ± 20% and All Enhancement Techniques: 66.67 ± 11.55%).

**Fig. 9:**
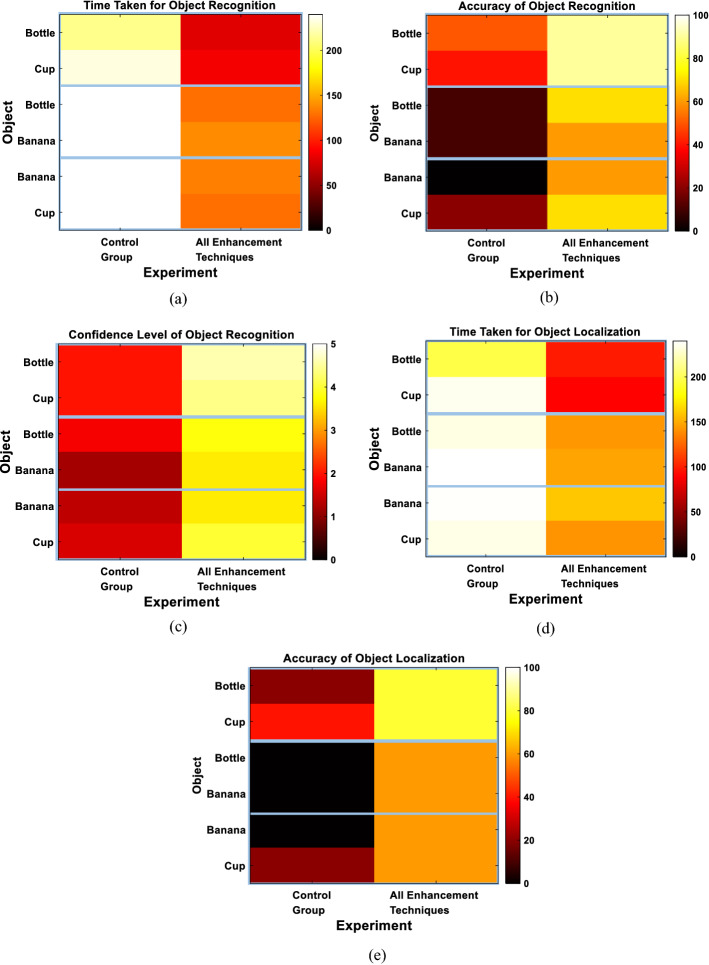
Multiple-object experiments results for each object. **a** Recognition time. **b** Recognition accuracy. **c** Confidence level. **d** Grasping attempt time. **e** Grasping accuracy. Blue rectangles in all figures represent the pairs of objects that were presented together

We finally evaluated the performance per each pair of presented objects. Figure 9 illustrates the outcome of each of the evaluation metrics for each object within each pair. The figure demonstrates that the recognition of the bottle and the cup was the easiest when they were presented together (Recognition Accuracy: 90% for both objects). Consistent results were also obtained for the localization of both objects compared to other objects (Localization Accuracy: 80%). This could be attributed to the significant difference in the shape between the two objects.

#### Navigation experiments

**Fig. 10:**
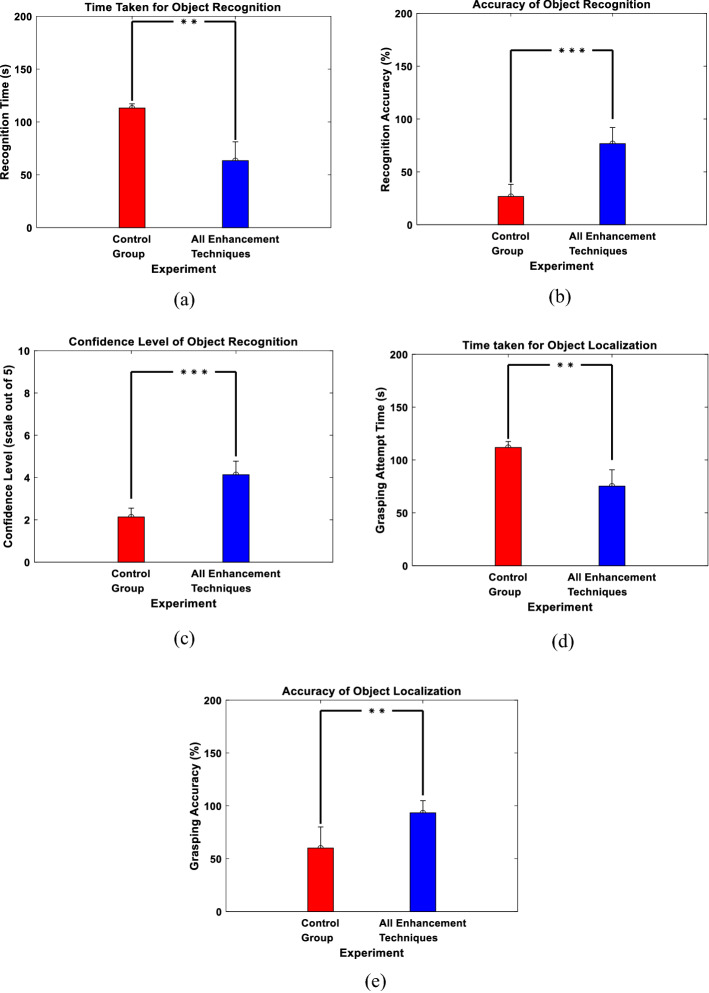
Navigation results. **a** Recognition time. **b** Recognition accuracy. **c** Confidence level. **d** Grasping attempt time. **e** Grasping accuracy. **P < 1e−04, ***P < 1e−07, two-sample Wilcoxon test

In the last set of experiments, we examined the ability of the participants to navigate freely in order to recognize and grasp an object. In terms of object recognition, Fig. 10a shows the time taken to correctly recognize objects. Consistent with the previous experiments, the experiment with all the enhancement techniques resulted in less time to recognize the objects compared to that of the control group experiment (Control Group: 126.4 ± 25.67 s and All Enhancement Techniques: 63.33 ± 17.81 s). Figure 10b confirms the same conclusion, where higher recognition accuracy was achieved when all the enhancement techniques were applied compared to that of the control group experiments (Control Group: 26.67 ± 11.55% and All Enhancement Techniques: 76.67 ± 15.28%). Figure 10c shows a similar result when measuring the confidence level of the participants (Control Group: 2.13 ± 0.42 and All Enhancement Techniques: 4.13 ± 0.64).

To demonstrate the results of the participants in object localization in this setup, Fig. 10d, e shows the time taken and the accuracy achieved by the participants to correctly grasp the object, respectively. Consistent with previous results, less amount of time and higher accuracy are achieved when using all the enhancement techniques compared to that of the control group experiments (Time Taken: Control Group: 111.8 ± 5.63 s and All Enhancement Techniques: 75.27 ± 15.45 s; Accuracy: Control Group: 60 ± 20% and All Enhancement Techniques: 93.33 ± 11.55%).

**Fig. 11:**
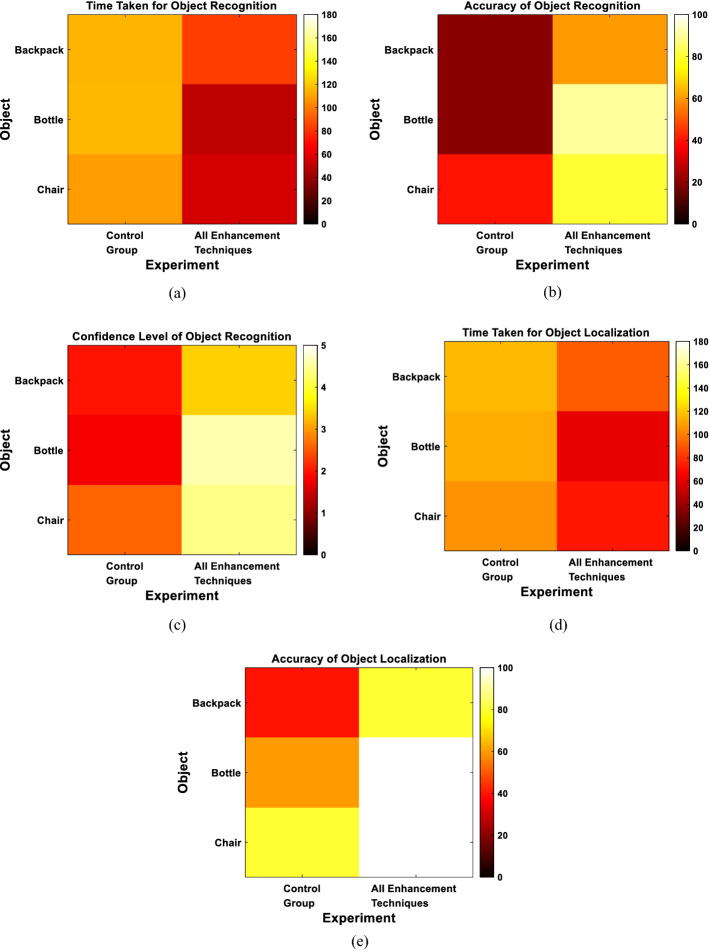
Navigation results for each object. **a** Recognition time. **b** Recognition accuracy. **c** Confidence level. **d** Grasping attempt time. **e** Grasping accuracy

Finally, to assess the performance for each object, Fig. 11 demonstrates the performance per each presented object. The figure shows that the recognition and localization of the bottle and the chair was relatively easier than those of the backpack. The chair and the bottle were easier to recognize and localize since the former has a very distinct shape with 4 legs for the chair while the latter has a simple shape of a vertical cylinder that could be easily recognized.

## Discussion

Visual prosthesis is considered a breakthrough that gives partial restoration of vision to blind patients to regain their confidence and independence. Although this might sound flawless, unfortunately, visual prostheses comprise a number of limitations that do not allow the complete perception of an image, which is far from what a normal sighted person sees. Some of the problems include the difficulty of correctly identifying and localizing objects.

We proposed a system for enhancing object recognition and localization through real-time mixed reality simulation. The 12 conducted experiments indicate that using clip art in place of the real object image significantly enhances the recognition of objects and that using edge enhancement and FAST corner detection significantly enhances objects localization. This could be attributed to the object simplification provided by using clip art and detail preservation provided by edge sharpening and FAST corner detection. Furthermore, the usage of the dropout handling approach enabled clearer representation of the objects giving the chance for accurate recognition of objects. The multiple-object experiments show the effect of using YOLO for objects’ detection to get the labels of the objects to retrieve the corresponding clip art representation. This enabled accurate selection of clip art object that identifies the real object’s identity. It is noteworthy that using clip art as opposed to providing an audio description of the viewed object is in alignment with the purpose of visual prostheses to partially restore vision without relying on other senses. Additionally, using clip art could be superior to using a textual description of the viewed object as this will require more processing time to display the detected word character by character. This is because it might be hard to recognize as a whole word through the typical limited resolution of visual prostheses. In addition, this might be problematic in case of having a long word since a real patient will not be able to memorize the sequence of letters they have already seen.

The navigation experiments show that allowing the participants to navigate while wearing the VR headset in real-time and perceiving the phosphene simulation of the physical floor, eases the ability to indicate that an object is placed on the floor and thus, the object localization is done easily. However, there could be some difficulty in recognizing the object due to the shadows of the light reflections in an indoor scene so the introduction of clip art with edge enhancement and FAST along with translating it to the location with minimum number of dropout locations, will facilitate the ability of the participants to correctly recognize an object. On the other hand, contour-based scene simplification via mobility was used in a real-world indoor scene using simulated prosthetic vision, where the representation of just the outline of an arbitrary object in addition to the discontinuities that are present in the contours hindered recognition accuracy, unlike the usage of clip art along with the other proposed enhancement techniques [19].

While the achieved results demonstrate the significance of the proposed enhancement technique, further enhancements could be utilized. For instance, the indoor room utilized in the experiments included other objects that were not part of the experiment, which sometimes confused some of the participants, thinking that those objects belong to the conducted experiment. In addition, in the multiple-object experiments, the utilization of more than two objects at a time could be performed to measure the recognition and localization accuracy when having a large number of objects that mimic a real-life perceived scene. Furthermore, YOLO can be retrained to cover a wider variety of other objects, both non-rigid and rigid objects, to be able to detect any object that a visual prostheses user might encounter. YOLO retraining has been proven to be efficient and has been able to provide remarkable accuracies when retrained using non-rigid objects such as clothing [21], protective clothing worn by workers to ensure their safety [22], hair and the upper body part of people [23]. Moreover, displaying the clip art in 3D and matching its perspective to the viewed object could allow better visualization of the orientation of the actual object. Furthermore, the performance of the approach could be also tested if each subject is presented with the outcome of different approaches in one experiment (with a smaller number of objects). This could help in examining the response of the same subject to each proposed approach. Finally, the proposed enhancement techniques could be examined in an outdoor environment to measure the efficacy of the proposed work and then examined in implanted visual prostheses users.

## Conclusion

Loss of vision affects the lives of millions of humans, affecting their confidence level and independence. While visual prostheses have represented a solution that could restore vision, at least partially, some challenges related to the resolution of the perceived image still need to be addressed. Accordingly, we proposed an image processing approach examined in real-time mixed reality simulation to enhance vision perceived via visual prostheses. We introduced four enhancement techniques which are clip art representation, edge enhancement, FAST and dropout handling. Twelve experiments were conducted in real-time in mixed reality to measure the ability of object recognition and localization. Three metrics were used in object recognition evaluation which were recognition time, recognition accuracy and confidence level. Moreover, two metrics were used to measure the object localization which were grasping attempt time denoting the attempt to localize the object and the grasping accuracy denoting the correctness of localization of the objects without using the sense of touch. The results demonstrate that the introduction of the four enhancement techniques gave the highest recognition accuracy, confidence level and grasping accuracy along with the least recognition time and grasping time. These four enhancement techniques showed that object recognition and localization will be enhanced when used in a real visual prosthetic system.

## Methods

### System overview

**Fig. 12:**
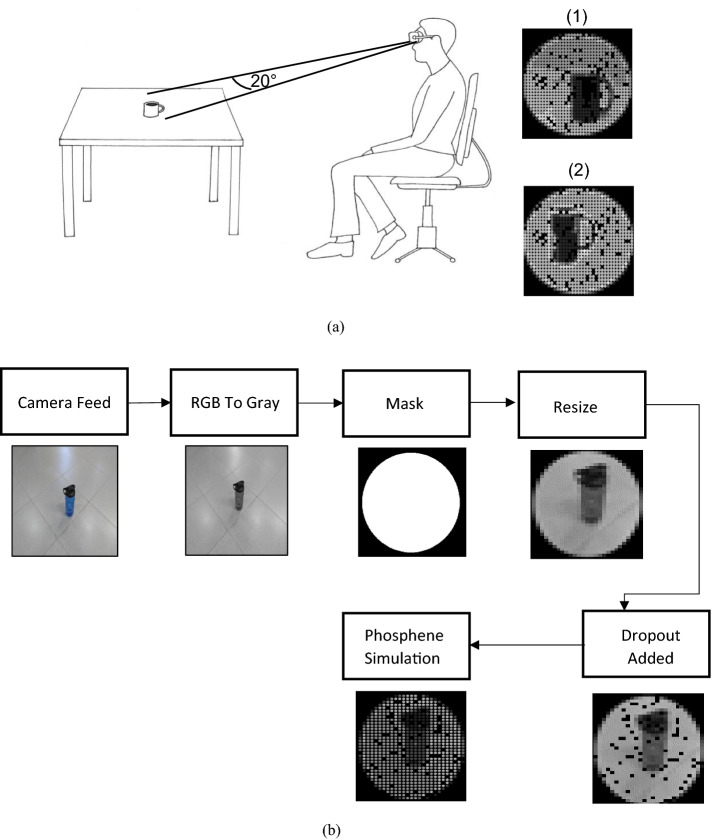
Displayed phosphene simulation. **a** Sample phosphene simulation perception in experiments: (1) before image enhancement, (2) after image enhancement. **b** Visual field adjustment in phosphene simulation

The proposed enhancement techniques were examined using simulation of prosthetic vision presented through a mixed reality setup as shown in Fig. 12a. The figure shows one sample object (a cup) that was presented to the subject. The camera in the setup takes a picture of the environment, where only 20° of the visual field is displayed inside the VR headset. The real object is displayed in its phosphene simulation allowing the participant to move freely to locate the object in its actual location. Phosphene simulation of the enhanced version of the object, including translating the enhanced image within the presented view to minimize the number of dropouts, is displayed to allow better recognition ability. The dropout locations were randomized across subjects. However, for each subject, the dropout locations remained fixed throughout the experiment to mimic the malfunctioning of specific electrodes in an implant.

The captured image is pre-processed through multiple stages as shown in Fig. 12b. First, an image is captured using the mobile camera that acts as the PC webcam. The image is then converted from RGB to grayscale to mimic the colors used by visual prosthetic users. Next, to simulate the visual field that visual prosthetic users encounter, a circular mask is applied. The radius of the mask reflects the 20° of the visual field given that the visual field in a prosthetic vision device is approximately 20° from the complete visual field [24]. Element-wise multiplication is performed between the mask and the pre-processed image so that only the part of the image residing inside the visual field is displayed. Furthermore, dropouts are added at random locations to mimic the malfunctioning of the electrodes at random positions.

### Phosphene simulation

The phosphene shape used was a round shape that matches the common form for phosphenes simulating the actual look of the phosphenes without any change in the stimulation amplitude and with ideal current set [25]. A squared grid was used in the phosphene simulation since it simulates the common grid used in visual prostheses [8]. Furthermore, the distance between each two successive phosphenes was set to zero. A dropout rate of 10% was used, where the dropout phosphene color was set to a black color [26]. We used 8 (3 bits) gray levels since the number of successfully distinguishable gray levels by real patients is in the range of 4–12 levels [27]. To map the initial 256 (8 bits) gray values to their corresponding gray value, a mapping scheme of 0, 36, 72, 108, 144, 180, 216 and 252 was utilized [28].

### Proposed enhancement techniques

#### Clip art representation

To enhance object recognition, we propose the utilization of a simplified version of an image, which is the clip art, to enable abstract representation of the image given the low spatial and radiometric resolutions [29]. The clip arts of the utilized objects in all of the experiments were selected, where the best shaped clip arts that easily identify an object are collected. The clip art size is adjusted to match that of the real object size. The clip art representation was mainly utilized to enhance the ability of the user to recognize the objects.

#### Edge enhancement

To enhance object localization, we use edge detection to emphasize the borders of the object of interest to facilitate its recognition. Canny edge detection was used to identify the edges in the object of interest where the edges in the vertical and horizontal directions are detected [30, 31]. The derivative of a Gaussian filter is used by the edge to determine the gradient. This technique detects both strong and weak edges using two thresholds and includes weak edges in the output if they are connected to strong edges. The Canny approach uses two thresholds, which makes it less susceptible to noise than the other methods and more likely to identify real weak edges. This thresholding is performed to the thinned edge magnitude image utilizing two edge strength thresholds named hysteresis. All pixels are candidates to be edge pixels where an edge pixel is a pixel that is above the low threshold of value 0.1 which can be connected to any arbitrary pixel above the high threshold of value 0.15 through a chain of edge pixels [31]. Moreover, non-maximal suppression is applied to thin the edges in which surviving candidate pixels are determined. Finally, the original grayscale image was added to the edge version of the object so that the edges in the object of interest will be sharpened.

#### Corner enhancement

For corner detection, we used Features from Accelerated Segment Test (FAST) for key points’ feature extraction, where a minimum accepted quality of corners of 0.1 and a minimum intensity of 0.2 were used [32]. FAST utilizes a circle comprising 16 pixels to determine corners from the candidate points. Every pixel is labeled from 1 to 16 clockwise. A corner pixel is a pixel where its intensity plus a threshold value *t*, is darker than a set of *N* pixels in the circle or its intensity minus a threshold value *t* of 0.2, is brighter than a set of *N* pixels. We utilized a value of *N* of 12 since that it is the most commonly used value so that the number of detected corners will be reasonable [33]. We utilized FAST algorithm to develop an interest point detector for utilization in real-time mixed reality simulation.

#### Dropout handling

Moreover, dropout handling technique was used to translate the object of interest to the best possible location within the visual field that includes the minimum amount of dropout. This is performed by applying convolution between the dropout matrix, which is the matrix that includes the dropout locations in the visual field, and the object of interest that is occupied inside a bounding box. Then, the location of the maximum value from the convolution operator is retrieved, which indicates the location with minimum dropout. Next, we subtract the midpoint of the object of interest from the location of the maximum value retrieved, and then translate the object of interest by the result of subtraction. This is performed after showing the phosphene simulation of the actual location of the object of interest to help in accurately identifying its location [16].

### Tools used

To create a real-time mixed reality setup, the following was performed: The Trinus VR application was used to display the phosphene simulation on a mobile screen that is placed inside the VR set (Electro Shinecon VR Box 3D headset, Dongguan Shinecon Industrial Co., Ltd, China). To allow the mobile camera to act as a PC webcam, a mobile application (IP Webcam) was used to enable capturing real-time images, sending them to the PC wirelessly to prepare the phosphene simulation and then, displaying the phosphene simulation on the mobile screen. The number of phosphenes used was 32 × 32 which is the threshold of scene recognition [34]. Moreover, a visual field of 20° was used to mimic the legal blindness threshold [35]. The process for each phosphene simulation takes 0.8 s, which is considered fast given the larger number of pixels used compared to that of a typical visual prosthetic device. The Trinus VR displayed the mask in the phosphene simulation as an ellipsoidal-like one, not circular. Therefore, to solve this issue, we manipulated the generated phosphenes to be circular by creating an ellipsoidal-like mask in the phosphene simulation so that a circular mask will be displayed using Trinus VR in the VR headset.

### Experimental setup

**Fig. 13:**
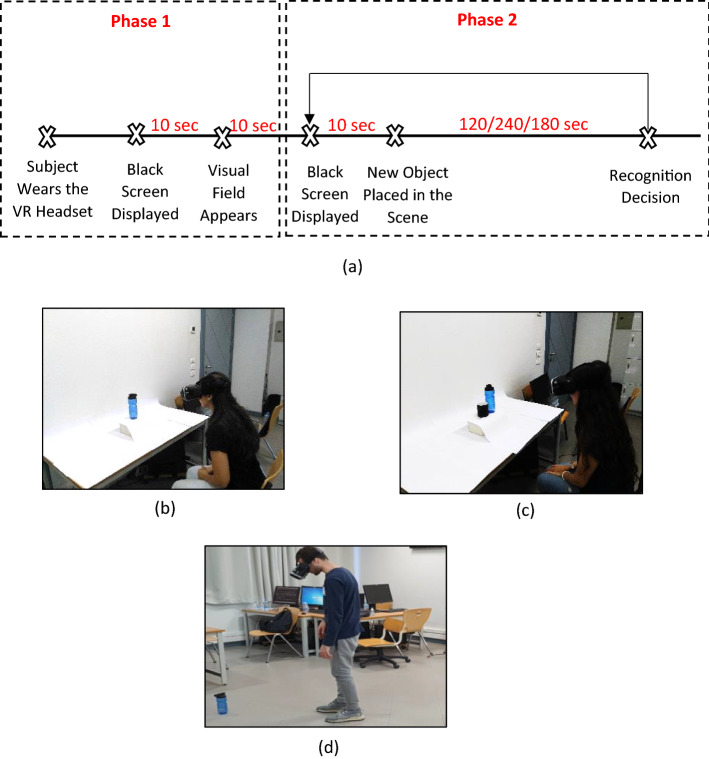
Experimental setup. **a** Experimental paradigm timeline. **b** Single-object experiment setup. **c** Multiple-object experiment setup. **d** Navigation experiment setup

All the experiments conducted were approved by the Faculty of Media Engineering and Technology, German University in Cairo from the ethical point of view. Twelve experiments were conducted on corrected vision/normally sighted subjects. Each of the 12 experiments comprised five participants giving a total of 60 participants involved in all of the experiments with a range of age from 19 to 36 years (23.37 ± 4.1 years) for both genders (34 males and 26 females). All the subjects signed a consent form indicating their confirmation to participate voluntarily in the experiments. The answers of the subjects were recorded through an audio recorder to avoid any human error in miscalculating the actual time for recognizing or localizing a certain object. Each of the participants was asked about their visual acuity and the reported visual acuity was recorded. However, visual acuity would have no impact on the results given the very low-resolution used in the phosphene simulations to mimic prosthetic vision resolution. Different subjects were involved in each experiment to avoid any learning effect that might arise due to prior knowledge of the displayed scene from a previous experiment. A demo experiment was presented to the participants before all the actual experiments to introduce the subjects to the phosphene simulation interpretation to be able to perceive the images used in the actual experiments in an easy manner. The experimental paradigm used in all of the conducted experiments is shown in Fig. 13a, where the subject first wears the headset, then there are two phases that the subjects pass through. In Phase 1, a black screen is displayed for 10 s to mimic the real environment that a real patient encounters before the visual prosthetic device is turned on and before being presented with any objects. Then, the phosphene simulation of the scene is presented to the subject before placing any object to explore the environment in front of the subject. In Phase 2, a black screen is displayed again for 10 s, but this time it acts as a cue that a new object is currently being placed in the scene. The subject is then allowed a maximum duration 120 s for the single-object recognition experiment and localization, 240 s for the multiple-object recognition and localization experiment, and 180 s for the navigation experiment. Phase 2 keeps repeating again as long as a new object is being placed in the scene. Three setups were used in the experiments. The first setup, shown in Fig. 13b, is for single-object recognition and localization where the participant is seated with the object being placed on a table with a white background to enhance the contrast between the background and the foreground. This setup comprised 8 experiments including an experiment on a control group. The second setup, shown in Fig. 13c, was for multiple objects recognition and localization. This setup comprised 2 experiments including an experiment on a control group. In the first two setups, the subjects were seated on a chair with the freedom of upper body movement such as getting closer to the object to allow zooming in the object or moving away from the object to allow zooming out of the object. The third setup, shown in Fig. 13d, was for objects recognition and localization while navigating in an indoor environment. This setup comprised 2 experiments including an experiment on a control group.

In all experimental setups, all participants were asked to name the object before attempting to grasp it. This is to ensure that the correct object recognition is only due to the ability of the participant to visually recognize the object, and not due to feeling the object when touching it during the grasp attempt. A set of questions was asked to the participants in all of the experiments as follows:

- Are you able to detect any object? (a) yes or (b) no.
- Describe the geometry of the shape you see: (a) rectangular, (b) curve-like or (c) other description.
- What is the shape?
- Can you determine its location? (a) yes or (b) no.
- How confident are you with your answer on a scale from 1 to 5, where 1 is the least confidence, and 5 is the highest confidence?

#### Single-object recognition and localization experiments

Five objects were used, which are cup, bottle, banana, car toy and laptop, in all of the eight experiments. The duration given for the participants for both object recognition and localization per one object was 120 s (i.e., 2 min). The participants wore the VR Box headset and were asked the aforementioned set of questions during the course of the experiment. The first experiment was the control group experiment which involved 2 males and 3 females. This group was presented with the phosphene simulation of the real object placed in front of them, with randomly generated dropouts added once at the beginning of the experiment for each subject and without using any enhancement technique. The second experiment was conducted on 4 males and 1 female, where the dropout handling technique was performed. This was in addition to the clip art representation of the real object, where the clip art object was resized and translated to the optimal location in the visual field. The third experiment involved 3 males and 2 females in which dropout handling was performed in addition to edge enhancement, applied to the real object image, using Canny edge detection technique. The fourth experiment involved 2 males and 3 females, where dropout handling was performed in addition to FAST corners detection to preserve the important details in the object. The fifth experiment comprised 4 males and 1 female where edge enhancement and FAST were applied to the real object without dropout handling. In the sixth experiment, 3 males and 2 females were involved in which clip art and edge enhancement were performed without dropout handling where the clip art is resized and translated to the location of the actual object in the visual field to maintain the actual location of the real object. The seventh experiment involved 4 males and 1 female where clip art and FAST were performed without dropout handling. Finally, the last experiment comprised 3 males and 2 females in which all enhancement techniques including clip art, edge enhancement, FAST and dropout handling are applied to the image. All the images displayed in all the experimental setups were represented in the phosphene simulation.

#### Multiple-object recognition and localization experiments

Three objects were taken in pairs at a time to be used in this type of experiments which are a bottle and a cup, a bottle and a banana, and a banana and a cup. The first experiment, involving 3 males and 2 females, was the control group experiment representing the phosphene simulation of the actual scene. The duration given for the participants for both object recognition and localization per one pair of objects was 240 s to match that of the duration used in the “All Enhancement Techniques” experiment. The second experiment, involving 2 males and 3 females, used all the enhancement techniques discussed before. Since the participant has the freedom to move his/her head to capture any of the two objects that are displayed at a time, You Only Look Once (YOLO) deep learning model is used to detect the object and get the corresponding clip art [36]. In the single-object experiments, only one object is displayed at a time and the order of displaying the objects is fixed across all participants, so the corresponding clip arts were easily determined without the need of waiting for YOLO to detect an already known object. Similar to the control group experiment, a duration of 240 s was used to be able to both recognize and localize each object per one pair of objects. The 240 s are utilized to give more chance for the participant to keep moving back and forth and left and right until being able to locate the full shape of an object so that YOLO is able to detect the object correctly and, therefore, the corresponding clip art will be retrieved. Once an object is detected by YOLO, the corresponding clip art is displayed in phosphene simulation. The best clip art shapes for the 80 classes used in the YOLO model were pre-determined, where each image was named based on the identity of the object. Then, the name (i.e., label) of the detected object is taken and compared to the prepared labels to find the corresponding clip art of the query real object. The clip art replaces the actual objects in the scene after applying edge enhancement, FAST and dropout handling. The dropout handling in this case was performed for each of the two bounding boxes of the detected objects separately. Objects were then translated one at a time. Each bounding box is translated to the ideal location (i.e., the location that fully occupies the object) in the visual field that contains the minimum number of dropouts.

#### Object recognition and localization during navigation experiments

Three objects were used in these experiments which are a backpack, a bottle and a chair. Since during navigation, visual prostheses users might encounter objects of different sizes, so we used an object of a big size (the chair), an object of a medium size (the backpack) and an object of a small size (the bottle). The duration given for the participants for both object recognition and localization per one object was 180 s (i.e., 3 min). Thus, one extra minute was added for each object to give the participant the chance to walk to the object location (i.e., 2 min + 1 min = 3 min per object) compared to that of the single-object experiments where no navigation was needed. This unifies the time across all other experimental setups. The first experiment, involving 3 males and 2 females, was the control group experiment without any enhancement applied to the actual image that is displayed in phosphene simulation. The second experiment, involving 2 males and 3 females, was the experiment that applied all the enhancement techniques. In both experiments, the subjects were asked to, first, move through the environment while looking at the floor, before any of the three objects is placed on the floor, to give them the chance to interpret how the floor would look like via phosphene simulation. This is to help them know that an object has been added by contrasting the difference in the perceived scene.

### Evaluation metrics

Five evaluation metrics were used in this article to evaluate the performance of the subjects in the experiments, where three of them were used for object recognition evaluation, while the other two were used for object localization evaluation. The evaluation metrics that were used in the object recognition are the recognition time measured in seconds, the recognition accuracy measured as the percentage of correctly recognized objects out of the total number of objects, and the confidence level on a scale from 1 to 5. The evaluation metrics that were used to measure the performance of the participants in objects localization were the grasping accuracy denoting the percentage of the correctly grasped objects with respect to the total number of objects, and the grasping attempt time measured in seconds. The grasping attempt time denotes the time at which the subject was extremely near to the object (i.e., within 10 cm from the object) or was able to directly grasp the object correctly without attempting to touch the object first to localize it. The 10-cm margin of error was utilized as a threshold for subjects who were relatively close to grasping the object, before mistakenly hitting the object leading to its fall. It should be noted that for the grasping accuracy, it was considered to be 100% if the object was correctly grasped. In case an object was not correctly grasped but the subject was 10 cm away from the object in their grasping attempt time, they were given a grasping accuracy of 0%.

## Data Availability

The datasets used and/or analyzed during the current study are available from the corresponding author on reasonable request.
